# Safety, Tolerability, Pharmacokinetics, and Acceptability of Oral and Long-Acting Cabotegravir in HIV-Negative Chinese Men

**DOI:** 10.1128/aac.02057-21

**Published:** 2022-03-15

**Authors:** Kelong Han, Paul Wannamaker, Hongzhou Lu, Biao Zhu, Meixia Wang, Melanie Paff, William R. Spreen, Susan L. Ford

**Affiliations:** a GlaxoSmithKline, Collegeville, Pennsylvania, USA; b ViiV Healthcare, Research Triangle Park, North Carolina, USA; c Shanghai Public Health Clinical Center, Fudan University, Shanghai, China; d The First Affiliated Hospital, School of Medicine, Zhejiang University, Hangzhou, China; e State Drug Clinical Trial Institution, Beijing Youan Hospital, Capital Medical University, Beijing, China; f GlaxoSmithKline, Research Triangle Park, North Carolina, USA

**Keywords:** cabotegravir, long-acting, Chinese, pharmacokinetics, HIV, preexposure prophylaxis

## Abstract

Long-acting (LA) cabotegravir demonstrated superior efficacy versus daily oral standard-of-care for HIV-1 preexposure prophylaxis. This phase 1 study assessed safety, tolerability, pharmacokinetics, and acceptability of cabotegravir in 47 HIV-negative adult Chinese men at low risk of acquiring HIV-1. Participants received once-daily oral cabotegravir 30 mg for 4 weeks and, after a 1-week washout, five 600-mg (3-mL) intramuscular cabotegravir LA injections at weeks 5, 9, 17, 25, and 33. Pharmacokinetic plasma samples were intensively collected on day 27 (*n* = 17) and sparsely collected before each injection until 56 weeks after final injection (*n* = 47). Cabotegravir LA injections were acceptable and well tolerated. Common adverse events included injection site pain, injection site swelling, and upper respiratory tract infection. No drug-related serious adverse events or deaths occurred. Mean cabotegravir concentration remained above 1.33 μg/mL (8× *in vitro* protein-adjusted concentration for 90% of the maximum inhibition of viral growth [PA-IC_90_]) before each injection and above 0.166 μg/mL (PA-IC_90_) for >32 weeks after final injection. Trough concentrations remained above PA-IC_90_ in nearly all participants and showed minimal accumulation. Noncompartmental pharmacokinetic analysis was performed. Geometric mean of terminal half-life was 1.89 and 47 days after oral and LA dosing, respectively. Cabotegravir concentrations were estimated to remain quantifiable for 48.7 weeks after final injection. Steady-state area under the concentration-time curve (AUC), peak concentration, trough concentration, terminal half-life, time to peak concentration, and apparent clearance after cabotegravir oral and LA dosing were similar to those estimated in non-Asian men in historical studies. These results support further clinical development of cabotegravir LA in China. (This study has been registered at ClinicalTrials.gov under registration no. NCT03422172.)

## INTRODUCTION

In 2020, approximately 38 million people were living with HIV globally, and an estimated 1.5 million people acquired HIV (1). Preventing transmission has long been important for controlling the AIDS epidemic, and preexposure prophylaxis (PrEP) has emerged as a key prevention strategy. The following two oral PrEP regimens were approved before the approval of cabotegravir long-acting (LA) for PrEP: tenofovir disoproxil fumarate plus emtricitabine (TDF/FTC) and tenofovir alafenamide plus emtricitabine (TAF/FTC) (2–5). Despite the availability of these daily oral PrEP options, rates of uptake and adherence have been suboptimal, especially among those at highest risk of acquiring HIV, thus limiting effectiveness (6–10).

The integrase strand transfer inhibitor cabotegravir is formulated as an LA injectable and offers an alternative to daily oral PrEP, requiring less frequent dosing and thereby possibly reducing challenges associated with adherence to a daily regimen. Cabotegravir LA 600 mg as a single agent administered intramuscularly (i.m.) every 8 weeks demonstrated superior efficacy to daily oral TDF/FTC for PrEP in cisgender men and transgender women who have sex with men (11) and in cisgender women (12) at high risk of acquiring HIV-1 through sexual transmission in two global, double-blind, double-dummy phase 3 studies including 7,790 participants (11, 12) and has been approved in the United States for use in adults and adolescents weighing ≥35 kg.

Rates of uptake and adherence to oral PrEP regimens among men who have sex with men in China have been low despite the increasing incidence of HIV-1 in this population as well as in China overall (13–18). However, cabotegravir has not been studied in China. It was considered important to evaluate cabotegravir safety and pharmacokinetics (PK) in Chinese participants because cabotegravir plasma PK may be impacted by intrinsic and extrinsic differences (e.g., body weight and habitus), possibly affecting absorption from the injection site and elimination from the systemic circulation.

Cabotegravir is a substrate of uridine 5′-diphosphosphate glucuronosyltransferase (UGT) and is primarily metabolized by UGT1A1 (19, 20). UGT1A1 polymorphisms identified in study participants that confer low predicted metabolic activity, specifically two copies of *28, *37, and *6 variants, were associated with a clinically nonsignificant increase in cabotegravir plasma concentrations after oral (28% to 50%) and LA administration (16% to 24%) compared with the UGT1A1 wild-type (*1) allele (21). Expression of UGT1A1 polymorphisms varies by ethnicity, with estimated UGT1A1*28 allele frequencies of 26% to 31% in Caucasians and 42% to 56% in African Americans; UGT1A1*36 and *37 alleles are primarily observed in African populations, with estimated frequencies of 3% to 10% and 2% to 7%, respectively; and the estimated frequency of the UGT1A1*6 allele is 23% among Chinese populations (22).

In addition, historical studies and population PK analysis have demonstrated that body weight and body mass index (BMI) are associated with cabotegravir plasma PK (23), while lower body weight and BMI have been observed in the Chinese population compared with Western populations (e.g., the United States) (24, 25). Lower body weight is associated with lower cabotegravir clearance and, therefore, higher plasma concentrations (23). Lower BMI is associated with faster cabotegravir LA absorption from the injection site and, therefore, higher peak concentrations, as observed in the phase 2 cabotegravir PrEP studies ECLAIR (ClinicalTrials.gov identifier, NCT02076178) (26, 27) and HPTN 077 (ClinicalTrials.gov identifier, NCT02178800) (28, 29). Despite the potential differences in LA absorption and elimination in the Chinese population resulting from lower body weight and/or lower BMI, the impact on cabotegravir plasma PK is expected to be minimal, with no dose adjustment required.

For these reasons, it is important to evaluate safety and plasma PK after cabotegravir oral and LA administration in the Chinese population. Herein, we report the safety, tolerability, PK, and acceptability of cabotegravir after oral and LA administration in HIV-negative adult Chinese men who are at low risk of HIV-1 acquisition (ClinicalTrials.gov identifier, NCT03422172).

## RESULTS

### Study population and baseline characteristics.

Of 64 individuals screened, 48 were enrolled, 47 completed the oral phase and received the first cabotegravir LA injection, 44 received the final injection, and 37 provided all safety and PK samples in the follow-up phase through week 89. In addition to the 37 participants who provided all safety and PK samples, five participants completed the final visit during the follow-up phase remotely without providing safety and PK samples due to the COVID-19 pandemic. The week 89 visit was delayed in 12 participants. One participant withdrew during the oral phase due to an adverse event (AE), and four withdrew during the injection phase (withdrawn consent, *n* = 3; lost to follow-up, *n* = 1). Of those enrolled, mean (range) age was 31 (20 to 53) years, mean (range) weight was 68.5 (47.5 to 91.5) kg, and mean (range) BMI was 23.3 (17.6 to 27.6) kg/m^2^.

### Safety.

All 47 participants in the injection phase reported at least one AE of injection site pain during the study. The most commonly reported AEs related to injection site reactions (ISRs) in ≥10% of participants were injection site pain (100%, *n* = 47; grade ≥2 severity, 38%, *n* = 18) and injection site swelling (47%, *n* = 22; grade ≥2 severity, 4%, *n* = 2). Among all 48 participants, the most commonly reported AEs not related to ISRs (≥10%) were upper respiratory tract infection (46%, *n* = 22; 3/22 had grade 2 events that occurred during the injection phase), pyrexia (21%, *n* = 10; 4/10 had grade 2 events that occurred 4 to 55 days postinjection and 9/10 had grade 1 events that occurred primarily 2 to 6 days postinjection with the exception of two events that occurred 146 and 338 days postinjection during the follow-up phase), cough (10%, *n* = 5), headache (10%, *n* = 5), and increased alanine aminotransferase (ALT) (10%, *n* = 5).

Grade 1 ALT elevation (1.25 to 2.5× upper limit of normal [ULN]) occurred in four participants (maximum observed individual ALT values of 60 to 80 IU/L). Grade 2 ALT elevation (2.5 to <5× ULN) occurred at week 65 (32 weeks after injection 5) in one participant (observed ALT value of 104 IU/L), who also experienced grade 1 ALT elevations at screening and weeks 9, 25, 33, 41, 77, and 89 (observed ALT values of 52 to 65 IU/L).

In the oral phase, no participants reported grade ≥ 3 AEs; six (13%) participants in the injection phase and eight (17%) in the follow-up phase reported grade ≥ 3 AEs, all of which were grade 3. Grade ≥2 drug-related AEs observed in >5% of participants in the injection phase were injection site pain (38%), pyrexia (6%), and upper respiratory tract infection (6%).

One participant in the oral phase experienced AEs that were considered related to treatment, which led to discontinuation of cabotegravir and study withdrawal. This participant experienced two nonserious AEs of nausea and vomiting (both grade 1) that resolved 1 day after onset. One participant experienced one serious AE (postprocedural hemorrhage after augmentation rhinoplasty that required hospitalization) during the injection phase; this event was not considered treatment related by the investigator, the event resolved, and the participant continued in the study. One participant was reported as having two grade 1 AEs of special interest (AESIs) of hepatic calcification and hepatic steatosis with concurrent cholelithiasis 16 days after receiving injection 2 that were considered treatment related; the participant completed the study, and the events were reported as not resolved. Additionally, during the injection phase, one participant experienced one grade 2 AESI of face swelling at the left cheek 16 days after receiving injection 3, which was considered treatment related; the event resolved within 3 days, and the participant completed the study. There were no drug-related serious AEs or deaths during the study.

### Pharmacokinetics.

**FIG 1:**
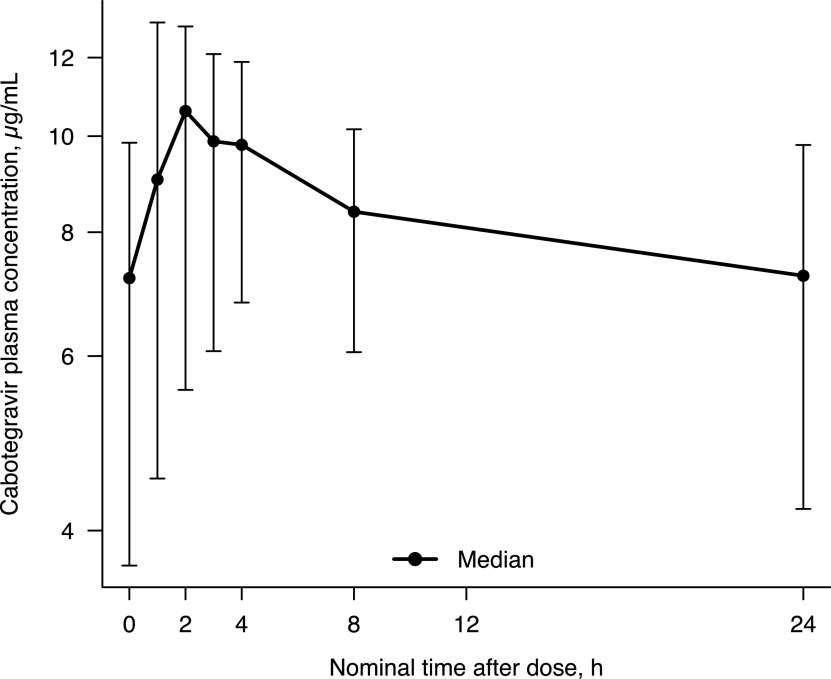
Cabotegravir plasma concentration-time profile on day 27 after oral cabotegravir 30 mg once daily. Error bars represent 5th and 95th percentiles.

Intensive PK data after oral dosing were collected in 17 participants (Fig. 1). After oral administration, cabotegravir was rapidly absorbed with a median time of occurrence of maximum observed concentration (*T*_max_) of 2 h postdose (Table 1). Subsequently, cabotegravir plasma concentration declined over time in a biphasic manner (Fig. 1). The geometric mean of terminal half-life (*t*_1/2_) was 1.89 days. Trough cabotegravir plasma concentrations were above 8× *in vitro* protein-adjusted concentration resulting in 90% of the maximum inhibition of viral growth for cabotegravir (PA-IC_90_) in all participants.

**TABLE 1:** Summary of repeat dose cabotegravir plasma PK parameters by route of administration^*a*^

PK parameter^*b*^	CAB oral (30 mg daily) on day 27 (*n *= 17)	CAB LA i.m. after injection 5 (wk 33) (*n *= 44)^*c*^
*C*_max_ (μg/mL)	10.4 (19.5) [9.46–11.5]	3.73 (66.1) [3.11–4.48]
AUC_0-_*_t_* (h · μg/mL)	191.1 (20.5) [172–212]	3443 (41.1) [3,049–3,887]^*d*^
Trough concentration (μg/mL)	6.81 (26.8) [5.95–7.79]	1.58 (43.1) [1.39–1.80]
*T*_max_, median (range) (h)	2.00 (0.983–4.00)	167 (0.00–765)
*t*_1/2_ (days)	1.89 (20.5) [1.70–2.09]	47.0 (71.2) [38.4–57.6]^*e*^
CL/*F* (L/h)	0.157 (20.5) [0.141–0.174]	0.174 (41.1) [0.154–0.197]^*d*^
*V_z_*/*F* (L)	10.3 (17.8) [9.36–11.2]	NR

a AUC_0-_*_t_*, area under the plasma concentration-time curve over the dosing interval; CAB, cabotegravir; CI, confidence interval; CL/*F*, apparent clearance after oral dosing; *C*_max_, maximum observed concentration; %CVb, between-participant coefficient of variation; i.m., intramuscular; LA, long-acting; NR, not reported; PK, pharmacokinetic(s); *t*_1/2_, terminal half-life; *T*_max_, time of occurrence of *C*_max_; *V_z_*/F, apparent volume of distribution after oral dosing.

b Data presents geometric mean (%CVb) [95% CI] unless otherwise noted.

c Valid PK parameters were derived for 44 participants unless otherwise noted.

d *n *= 43.

e *n *= 41.

**FIG 2:**
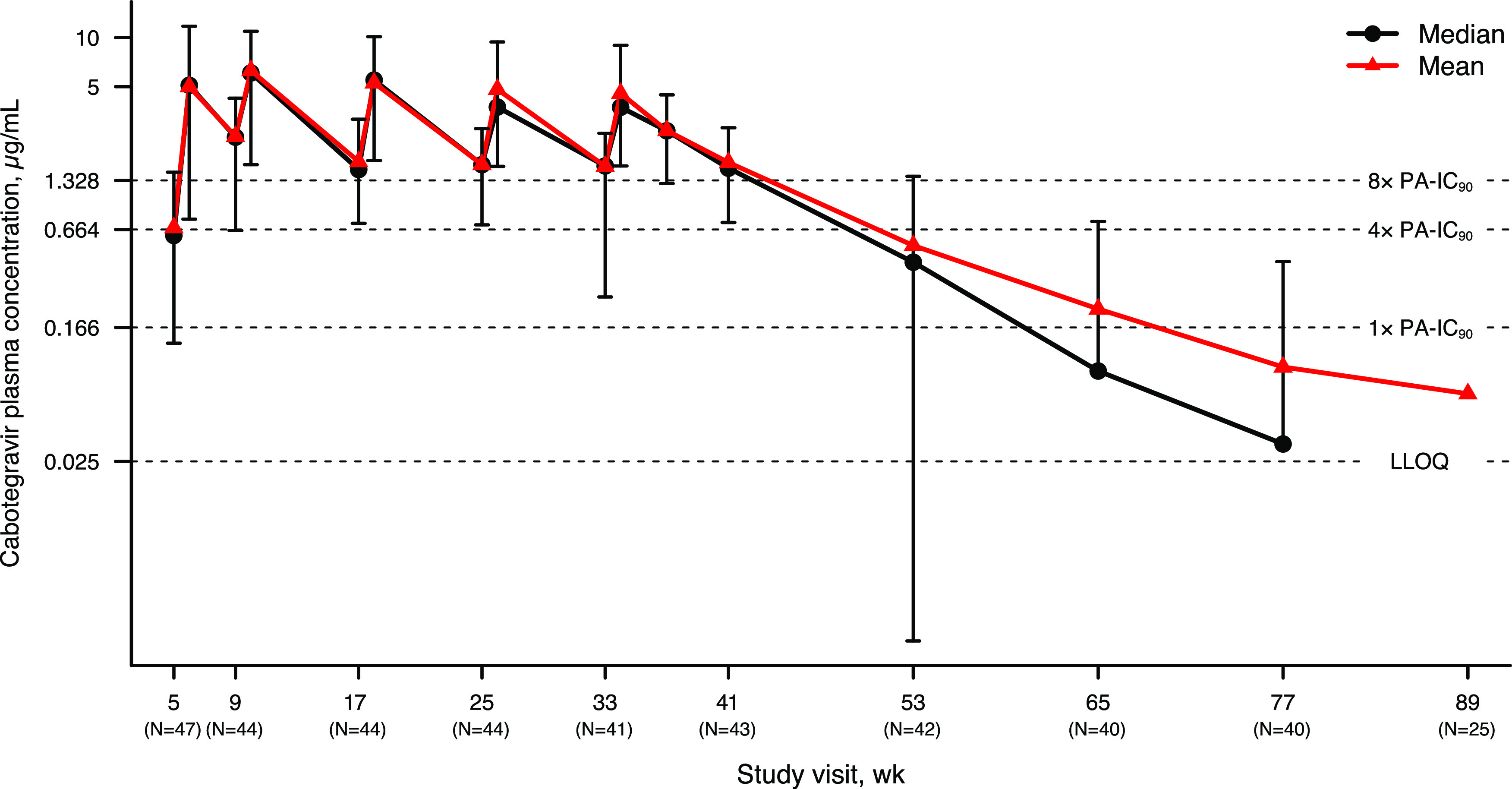
Cabotegravir plasma concentration-time profile after five cabotegravir LA injections (first injection was at week 5; final injection was at week 33) through week 89. Error bars represent 5th and 95th percentiles. Numbers in parentheses on *x* axis represent the numbers of participants with cabotegravir concentrations at trough and concentrations in the follow-up phase. Nonquantifiable concentrations were imputed as zero for the purpose of calculating statistics. LLOQ, lower limit of quantitation; PA-IC_90_, *in vitro* protein-adjusted concentration resulting in 90% of the maximum inhibition of viral growth for cabotegravir. The week 89 visit was delayed in 12 participants, and therefore, 25/37 participants who completed the week 89 visit were considered within the visit window. Median at week 89 and the 5th percentile beyond week 53 were zero and, therefore, could not be displayed.

**FIG 3:**
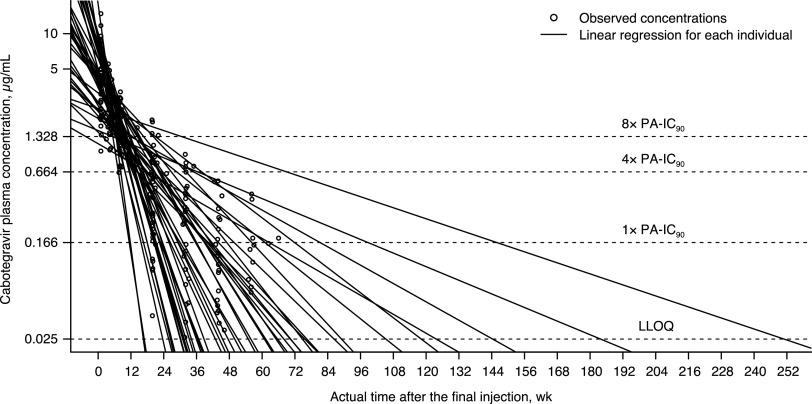
Individual cabotegravir terminal concentration-time profile with log-linear regression after the final injection. Linear regression was applied to fit the log-transformed cabotegravir plasma concentration data over actual sampling time after the final injection in each participant (solid line) and to calculate the time from the final injection to the time when cabotegravir plasma concentration decreased to LLOQ. PA-IC_90_ = 0.166 μg/mL. LLOQ, lower limit of quantitation; PA-IC_90_, *in vitro* protein-adjusted concentration resulting in 90% of the maximum inhibition of viral growth for cabotegravir. All 44 participants were included in this analysis, regardless of whether the week 89 visit was delayed or not.

Sparse PK data after LA dosing are shown in Fig. 2. After LA administration, the plasma cabotegravir concentration-time profile was characterized by a slow absorption phase and a prolonged terminal elimination phase. Median *T*_max_ was 7 days postdose (Table 1). Trough cabotegravir plasma concentrations were similar after injections 2 to 5, indicating minimal accumulation in cabotegravir plasma concentrations after the second injection. The geometric mean of the terminal *t*_1/2_ after the final injection (fifth injection), including the follow-up phase, was 47 days. The median time from the final injection to the time when cabotegravir plasma concentration decreased to lower limit of quantitation (LLOQ) was calculated to be 48.7 weeks (Fig. 3).

Geometric mean of steady-state maximum observed concentration (*C*_max_) after oral administration (10.4 μg/mL) was 2.8-fold of that after LA administration (3.7 μg/mL), consistent with the 2.8-fold higher daily oral dose of 30 mg compared with the estimated injected average daily dose of 10.7 mg (600 mg divided by 56 days). Similarly, when normalized to daily dose, steady-state area under the plasma concentration-time curve over the dosing interval (AUC_0-_*_t_*) after oral administration (6.37 h · μg/mL per day) was similar to that after LA administration (5.74 h · μg/mL per day). Mean and median cabotegravir plasma concentrations were maintained above 1.33 μg/mL (8× PA-IC_90_), and the lower boundary of the standard deviation (SD) exceeded 4× PA-IC_90_ at all visits in the injection phase through week 41, the trough after the fifth injection at week 33.

**FIG 4:**
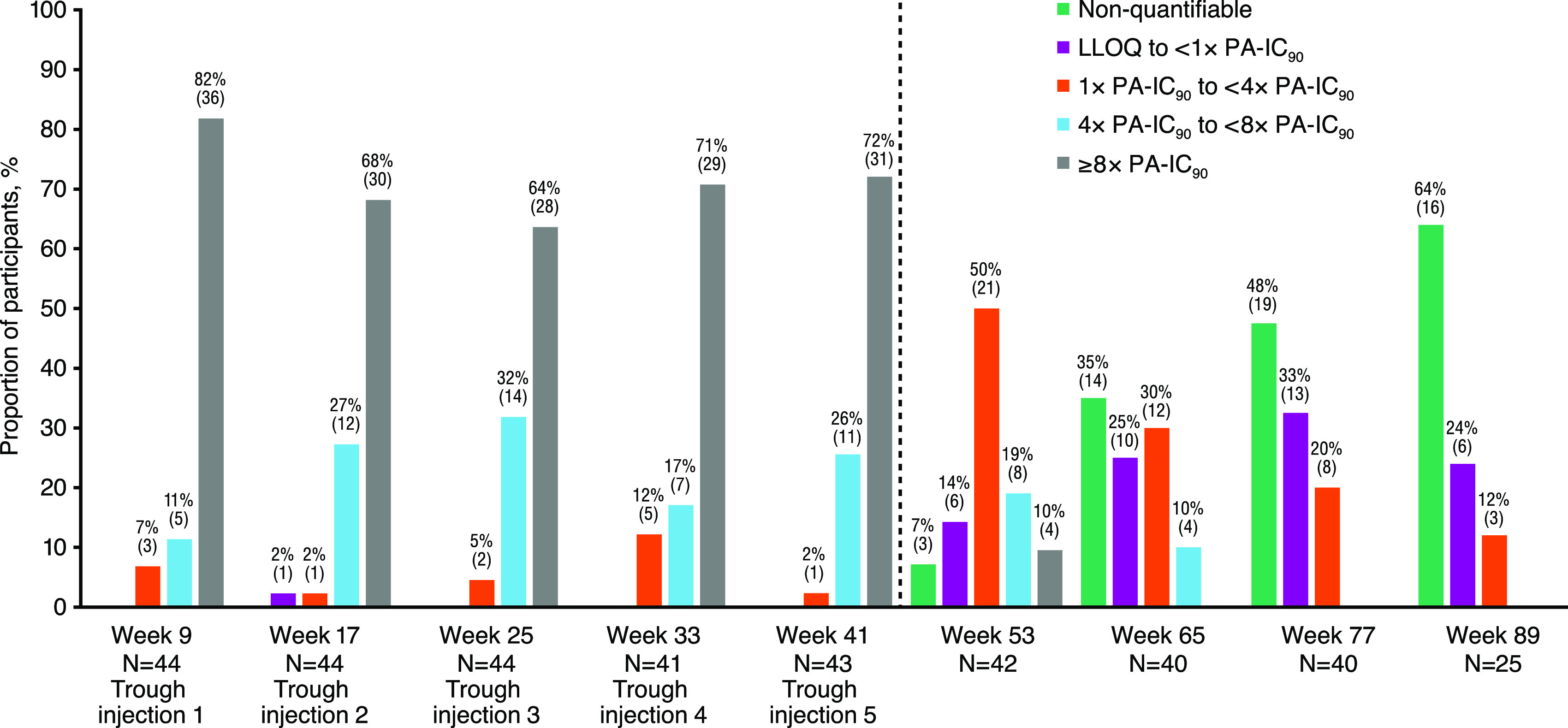
Proportion of participants with cabotegravir at trough and concentrations in follow-up phase above various thresholds by visit. PA-IC_90_ = 0.166 μg/mL. LLOQ, lower limit of quantitation; PA-IC_90_, *in vitro* protein-adjusted concentration resulting in 90% of the maximum inhibition of viral growth for cabotegravir. The first injection was at week 5 and the final injection was at week 33. The week 89 visit was delayed in 12 participants, and therefore, 25/37 participants who completed the week 89 visit were considered within the visit window. The number in parentheses represents the number of participants.

The proportion of participants with cabotegravir at trough and concentrations in the follow-up phase above various thresholds by visit are shown in Fig. 4. During the injection phase, plasma concentration at trough was above PA-IC_90_ (0.166 μg/mL) across visits in all participants, except for one participant with a concentration of <1× PA-IC_90_ at week 17 before injection 3 (Fig. 4). Plasma trough was above 4× and 8× PA-IC_90_ in >87% and >63% of all participants, respectively. During the follow-up phase, at week 53 (i.e., 20 weeks after the final injection), 29% (12/42) of participants had cabotegravir concentrations of >4× PA-IC_90_ and 50% (21/42) between 1× and <4× PA-IC_90_. At week 77 (44 weeks after the final injection), 20% (8/40) of participants had cabotegravir concentrations ranging from 1× to <4× PA-IC_90_, 33% (13/40) between LLOQ and 1× PA-IC_90_, and 48% (19/40) below LLOQ. At week 89 (final visit, 56 weeks after the final injection), 64% (16/25) of participants had concentrations below LLOQ, 24% (6/25) between LLOQ and 1× PA-IC_90_, and 12% (3/25) between 1 and <4× PA-IC_90_ (Fig. 4). The week 89 visit was delayed in 12 participants; therefore, 25/37 participants had values within the week 89 study window. Of the 12 participants with a delayed week 89 visit, the final visit occurred 58 to 66 weeks after the final injection (instead of 56 weeks), and 10/12 (83%) of the final cabotegravir plasma concentrations were nonquantifiable.

One participant acquired HIV-1 at week 89. For this participant, cabotegravir plasma concentration was 0.0495 μg/mL at week 65 and not quantifiable at weeks 77 and 89. Thus, cabotegravir plasma concentration was below the PA-IC_90_ of 0.166 μg/mL for ≥6 months before the confirmed HIV-1 diagnosis.

### Tolerability and acceptability.

Overall, 84% (*n* = 38) and 87% (*n* = 39) of 45 participants (score of 1 to 3 on the HIV-Prevention Treatment Satisfaction Questionnaire, change version) reported being willing to recommend the medication and were willing to continue with the study medication, respectively. Assessment of the tolerability of cabotegravir LA injections showed that 91% (*n* = 41) of participants were satisfied with side effects, and 78% (*n* = 35) tolerated the pain/discomfort. Most participants (98%, *n* = 44) were satisfied with their current treatment to prevent HIV (score of 1 to 3), with a median total score (across all 13 items) of 23. Most participants (83%, 40/48) considered using cabotegravir LA for HIV prevention in the future.

## DISCUSSION

The primary objectives of this study were to evaluate the safety, tolerability, and PK of cabotegravir LA 600 mg administered i.m. every 4 weeks for two doses followed by every 8 weeks for three doses in HIV-negative Chinese men. The safety results observed in the present study are consistent with those reported from other cabotegravir LA studies (11, 26–31). The mild-to-moderate nature of the ISRs seen in the present study has also been observed in previous studies of HIV-negative volunteers and trial participants at high risk of acquiring HIV (32, 33). Cabotegravir PK results from this study were similar to those in non-Asian male participants in the HPTN 077 study (Table 2) (28, 29).

**TABLE 2:** Comparison of CAB LA PK parameters after the final injection between adult Chinese male participants and non-Asian male participants in HPTN 077^*a*^

PK parameter^*b*^	Chinese male participants in present study (*n* = 44)	Non-Asian male participants in HPTN 077 (*n* = 18)^*c*^
AUC_0-_*_t_* (h · μg/mL)	143 (127–162)^*d*^	163
*C*_max_ (μg/mL)	3.73 (3.11–4.48)	3.82
Trough concentration (μg/mL)	1.58 (1.39–1.80)^*d*^	1.68
*t*_1/2_ (days)	47.0 (38.4–57.6)^*e*^	45.3
Estimated time to LLOQ, median (range) [IQR] (wks)	48.7 (16.5–251.0) [31.9–71.5]	43.7 (20.4–152.5) [31.1–66.6]

a AUC_0-_*_t_*, area under the plasma concentration-time curve over the dosing interval; CAB, cabotegravir; CI, confidence interval; *C*_max_, maximum observed concentration; LA, long-acting; LLOQ, lower limit of quantitation; PK, pharmacokinetic(s); *t*_1/2_, terminal half-life. All values calculated based on the final injection.

b Data presents geometric mean (95% CI) unless otherwise noted.

c Only published data for non-Asian male participants in HPTN 077 are listed. Source data are unavailable; therefore, some 95% CIs are unavailable (30, 36).

d *n *= 43.

e *n *= 41.

The safety and PK results from this study were compared with those from HPTN 077 because the studies were conducted in a similar fashion as follows: identical dose level and dosing schedules, PK samples collected for an extended period (up to week 109 in cohort 2 of the HPTN 077 study) after the final injection, enrollment of HIV-negative participants, and use of the same cabotegravir LA formulation (28, 29).

The safety profile of cabotegravir LA observed in this study was similar to the HPTN 077 study (29). Grade ≥2 ISR AEs observed in this study occurred at a similar event rate in the HPTN 077 study. HPTN 077 was a placebo-controlled study and reported that the only grade ≥2 AE that was more common in the cabotegravir group compared with the placebo group was ISRs (*P *< 0.001). Although AEs of upper respiratory tract infection were common in this study, only 6% of participants reported grade ≥2 AEs of upper respiratory tract infection, a lower rate than was reported in the cabotegravir group in the HPTN 077 study (23%).

The proportions of participants with cabotegravir trough concentrations above and below various thresholds (i.e., LLOQ, 1× PA-IC_90_, 4× PA-IC_90_, and 8× PA-IC_90_) at each of the five injection visits in this study were similar to the proportions of non-Asian male participants in HPTN 077 who received the same cabotegravir dosing regimen. Cabotegravir LA PK parameter estimates were also similar. Additionally, the proportions of participants with cabotegravir concentrations above and below various thresholds (LLOQ, 1× PA-IC_90_, 4× PA-IC_90_, and 8× PA-IC_90_) at each scheduled visit after the final injection in this study were similar to the proportions of non-Asian male participants in cohort 2 of the HPTN 077 study who received the same cabotegravir dosing regimen.

Cabotegravir plasma PK parameters and exposure in this study were consistent with those observed in other cabotegravir studies (23, 27–31). The geometric mean (95% confidence interval [CI]) trough concentration at week 41 (trough for the week 33 injection) in this study was 1.58 μg/mL (1.39 to 1.80), similar to the geometric mean (95% CI) at trough of 1.58 μg/mL (1.49 to 1.68) at week 48 in participants who had no prior exposure to cabotegravir and received cabotegravir 600 mg every 8 weeks (*n* = 217) in the phase 3 ATLAS-2M study (ClinicalTrials.gov identifier, NCT03299049) (unpublished data). The geometric mean of the terminal slope after the final injection observed in this study was 0.000614 h^−1^, similar to the population estimate of terminal slope of 0.000733 h^−1^ for the 1,223 male participants in the final population PK model (23).

In the injection phase of this study, peak arithmetic mean cabotegravir plasma concentrations occurred within 1 week of each injection, and mean concentration at trough remained above 1.62 μg/mL throughout the injection phase. Target cabotegravir trough concentrations were achieved consistently throughout the injection phase (Fig. 4): PA-IC_90_ (0.166 μg/mL) was achieved in ≥98% of participants, 4× PA-IC_90_ (0.664 μg/mL) in ≥88% of participants, and 8× PA-IC_90_ (1.328 μg/mL) in ≥64% of participants. Concentrations remained above PA-IC_90_ in all but one participant throughout dosing during the injection phase. After the final injection was administered at week 33 (injection 5), mean cabotegravir plasma concentrations were maintained above 0.166 μg/mL (PA-IC_90_) until between weeks 65 and 77 and above 0.664 μg/mL (4× PA-IC_90_) until week 53.

The prolonged terminal half-life of cabotegravir LA and the persistence of plasma concentrations after the final injection are both an advantage and a limitation. The prolonged terminal half-life of cabotegravir LA allows for the convenience of infrequent injections, such as once every 8 weeks, and could potentially maintain efficacy for a prolonged period of time after interruption or discontinuation of injections. However, upon discontinuation of cabotegravir LA injections, cabotegravir plasma concentration was predicted to remain below preventive or therapeutic thresholds but was quantifiable for years (Fig. 3), potentially increasing the risk of development of drug resistance.

Most participants were satisfied with cabotegravir LA as an HIV prevention modality, felt that the LA regimen was effective and acceptable compared with the oral cabotegravir regimen, and would plan to use cabotegravir LA for HIV prevention in the future if needed, indicating that cabotegravir LA was well tolerated. Although injection site pain was common, results suggest that participants experienced a high level of overall satisfaction and preference for LA injectable PrEP and reported convenience, flexibility, and ease of use as important factors in their satisfaction. These results are consistent with those from the phase 2 ECLAIR trial (34), in which participants also reported high levels of satisfaction, preference for cabotegravir LA over oral regimens, and a willingness to continue and recommend cabotegravir LA using the Study Medication Satisfaction Questionnaire (35).

Limitations of this study include the small sample size, which may have limited the detection of rare AEs. It should also be noted that ISRs and health outcome assessments were self-reported and, therefore, could have been biased by individual interpretation. Additionally, polymorphisms in UGT1A1 metabolizing enzymes that may have contributed to the higher observed concentrations were not assessed. Finally, this study only enrolled Chinese participants who were male at birth. Female at birth and/or transgender participants in pivotal cabotegravir LA PrEP studies were enrolled at global clinical sites, including in North and South America, Africa, and Asia (Thailand and Vietnam); however, none were from China, and further evaluation of cabotegravir LA for PrEP in Chinese individuals may be warranted.

In conclusion, oral cabotegravir 30-mg tablets administered once daily for 4 weeks and cabotegravir 600-mg LA injections (3-mL single intramuscular injections into the gluteus medius) administered every 8 weeks, with the first two injections given 4 weeks apart, were studied in HIV-negative Chinese men. These dosing regimens demonstrated a favorable safety profile, tolerability, and acceptability in a Chinese population, similar to those observed in historical cabotegravir studies. Cabotegravir trough concentration was maintained above various *in vitro* thresholds during cabotegravir LA dosing. Cabotegravir plasma PK exposure and PK parameters in Chinese men after oral and LA cabotegravir dosing were similar to those estimated in men from non-Asian regions in historical studies. These results support further clinical development of cabotegravir LA in China.

## MATERIALS AND METHODS

### Study design.

**FIG 5:**
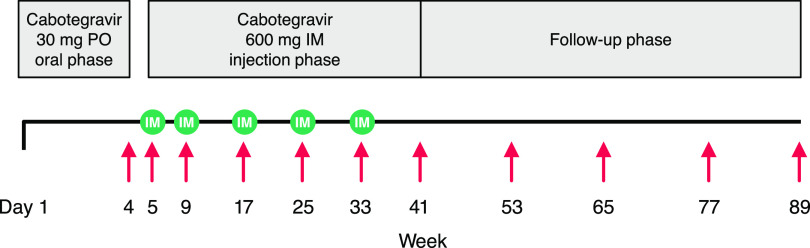
Study design. Red arrows denote PK sampling (intensive PK sampling occurred in only 17 participants at week 4); green circles denote injection. IM, intramuscular; PK, pharmacokinetic; PO, daily oral.

This was an open-label, nonrandomized, single-arm, multicenter phase 1 study conducted in China to evaluate the safety, tolerability, PK, and acceptability of cabotegravir after oral and LA intramuscular administration in HIV-negative adult male Chinese participants at low risk of HIV-1 acquisition (ClinicalTrials.gov identifier, NCT03422172). As shown in Fig. 5, eligible participants first received oral cabotegravir (30-mg tablets) once daily for 4 weeks during the oral phase (i.e., weeks 1 to 4) to assess individual tolerability before receiving cabotegravir LA injections. After a 1-week washout upon completion of the oral phase, given the oral elimination *t*_1/2_ of 35 to 42 h observed in historical cabotegravir studies, participants received five cabotegravir LA 600-mg injections (3-mL single intramuscular injection into the gluteus medius) once every 8 weeks with the first two injections given 4 weeks apart (i.e., weeks 5, 9, 17, 25, and 33).

This study was conducted in accordance with International Conference for Harmonization of Technical Requirements for Registration of Pharmaceuticals for Human Use Good Clinical Practice and applicable country-specific requirements, applicable laws and regulations, and consensus ethical principles derived from international guidelines including the Declaration of Helsinki and Council for International Organizations of Medical Sciences International Ethical Guidelines. Written informed consent was obtained from each participant before the performance of any study-specific procedures.

### Study population.

This study recruited HIV-negative Chinese participants aged 18 to 65 years who were male at birth, had both a nonreactive point-of-care HIV test and undetectable HIV-1 RNA at screening, and were at low risk of acquiring HIV-1. Participants were required to agree to appropriate use of contraceptive measures if participating in heterosexual/vaginal intercourse (participants were counseled on safer sexual practices, including use of effective barrier methods such as male condoms to reduce risk of sexually transmitted infections). Participants were excluded from the study if they had received antiretroviral therapy in the past 30 days, had current or chronic history of liver disease or known hepatic or biliary abnormalities, history of cardiac disease, active skin disease or disorder, high risk of seizures, other medical conditions (including psychiatric conditions) that would interfere with the ability to complete study procedures, or were determined to pose a significant suicide risk. Participants were also excluded if they were at high risk of acquiring HIV-1, which was defined as including one or more of the following: being the negative partner in an HIV-serodiscordant couple where the partner with HIV is not suppressed, exchanging sex for goods or money, engaging in any condomless anal intercourse in the past 6 months, having >5 male or female sexual partners in the past 6 months, having a sexually transmitted infection in the past 6 months, or engaging in any other behavior assessed by the investigator as “high risk.”

### Procedures.

Adverse events were assessed at day 1 and all study visits thereafter; vital signs were assessed at screening, day 1, week 5, and all visits thereafter. Clinical laboratory testing was performed at screening; day 1; and weeks 4, 5, 6, 9, 17, 25, 33, 41, and 89. Point-of-care HIV testing was performed at screening, day 1, each study visit before each injection, 8 weeks after the final injection, and week 89.

To characterize cabotegravir oral PK, a subgroup of participants volunteered to take part in the intensive PK assessment during the oral phase and underwent 24-h serial PK blood sampling at predose and 1, 2, 3, 4, 8, and 24 h postdose after the 27th oral dose on days 27 and 28. Pharmacokinetic sampling during the injection phase and follow-up phase was conducted at every visit shown in Fig. 5: before each injection (weeks 5, 9, 17, 25, and 33), 1 week after each injection (weeks 6, 10, 18, 26, and 34), 4 and 8 weeks after the final injection (weeks 37 and 41), and then every 12 weeks during the long-term follow-up phase (weeks 53, 66, 77, and 89).

Plasma samples were analyzed for cabotegravir concentration using a validated analytical method (28) based on protein precipitation, followed by high-performance liquid chromatography with tandem mass spectrometry analysis. The assay has a 1,000-fold linear range with an LLOQ of 0.025 μg/mL and an upper limit of quantitation of 25 μg/mL.

### Study objectives and endpoints.

The primary study objectives were evaluation of the safety and tolerability of cabotegravir LA throughout the injection phase and plasma PK of cabotegravir after repeat oral and LA administration. Safety and tolerability endpoints included evaluation of AEs, clinical laboratory tests, study withdrawals due to AEs, vital signs assessments, and ISRs. Plasma cabotegravir PK parameters included concentration at the end of the dosing interval, AUC_0-*t*_, *C*_max_, *T*_max_, apparent clearance after oral dosing (CL/*F*), apparent volume of distribution after oral dosing (*V_z_*/*F*), terminal *t*_1/2_, and terminal elimination phase rate constant (λz).

Pharmacokinetic targets of cabotegravir trough concentration for preventive efficacy used in this study were selected based on macaque models using a rectal or vaginal simian/human immunodeficiency virus (SHIV) challenge (36, 37): PA-IC_90_ (0.166 μg/mL) should be achieved in 95% of participants, 4× PA-IC_90_ (0.664 μg/mL) in 80% of participants, and 8× PA-IC_90_ (1.328 μg/mL) in 50% of participants (i.e., the target of median cabotegravir trough concentration is 1.328 μg/mL). The PK targets selected in this study were consistent with those in other cabotegravir PrEP studies (11, 12, 26–29).

Tolerability and acceptability of cabotegravir LA injections were key secondary objectives. The 13-item HIV-Prevention Treatment Satisfaction Questionnaire, change version score was used to assess tolerability via measurement of the experience of prevention treatment using a scale from 3 (much more satisfied/effective/convenient/flexible/likely to recommend the treatment/likely to speak well of the treatment/easier now) to −3 (much less satisfied/effective/convenient/flexible/likely to recommend the treatment/likely to speak well of the treatment/easier now). In addition, participants were asked if they would consider using cabotegravir LA for HIV prevention in the future at their week 41 visit or at their withdrawal visit, whichever occurred first. The proportion of participants who would consider using cabotegravir LA for HIV prevention in the future was calculated as an assessment of acceptability.

### Statistical analysis.

Participants who had at least one postdose quantifiable cabotegravir plasma concentration were included in the analysis. Nonquantifiable concentrations were not imputed for estimating PK parameters. Pharmacokinetic parameters were estimated from concentration-time data using noncompartmental methods with Phoenix WinNonlin version 8.0 (Certara, Princeton, NJ), and results are presented as descriptive statistics. Linear regression was applied to fit the log-transformed cabotegravir plasma concentration data in the terminal phase over actual sampling time after the final injection in each participant (the same method to estimate the terminal *t*_1/2_ by noncompartmental analysis). The time from the final injection to the time when cabotegravir plasma concentration decreased to LLOQ was calculated by extrapolating the linear regression beyond the observed concentrations. Safety and tolerability results are summarized using descriptive statistics.

### Data availability.

Anonymized individual participant data and study documents can be requested for further research from www.clinicalstudydatarequest.com.

## References

[1] UNAIDS. 2021. Fact sheet – World AIDS Day 2021. Global HIV statistics. UNAIDS, Geneva, Switzerland. https://www.unaids.org/sites/default/files/media_asset/UNAIDS_FactSheet_en.pdf.

[2] Baeten JM, Haberer JE, Liu AY, Sista N. 2013. Preexposure prophylaxis for HIV prevention: where have we been and where are we going? J Acquir Immune Defic Syndr 63:S122–S129. 10.1097/QAI.0b013e3182986f69. 23764623 PMC3710117

[3] Gilead Sciences, Inc. 2020. Truvada: prescribing information. Gilead Sciences, Inc, South San Francisco, CA.

[4] Gilead Sciences, Inc. 2021. Descovy: prescribing information. Gilead Sciences, Inc, South San Francisco, CA.

[5] National Institutes of Health. 2021. The basics of HIV prevention. National Institutes of Health, Bethesda, MD. https://hivinfo.nih.gov/understanding-hiv/fact-sheets/basics-hiv-prevention.

[6] Haberer JE, Bangsberg DR, Baeten JM, Curran K, Koechlin F, Amico KR, Anderson P, Mugo N, Venter F, Goicochea P, Caceres C, O'Reilly K. 2015. Defining success with HIV pre-exposure prophylaxis: a prevention-effective adherence paradigm. AIDS 29:1277–1285. 10.1097/QAD.0000000000000647. 26103095 PMC4480436

[7] Hess KL, Hu X, Lansky A, Mermin J, Hall HI. 2017. Lifetime risk of a diagnosis of HIV infection in the United States. Ann Epidemiol 27:238–243. 10.1016/j.annepidem.2017.02.003. 28325538 PMC5524204

[8] Van Damme L, Corneli A, Ahmed K, Agot K, Lombaard J, Kapiga S, Malahleha M, Owino F, Manongi R, Onyango J, Temu L, Monedi MC, Mak'Oketch P, Makanda M, Reblin I, Makatu SE, Saylor L, Kiernan H, Kirkendale S, Wong C, Grant R, Kashuba A, Nanda K, Mandala J, Fransen K, Deese J, Crucitti T, Mastro TD, Taylor D, for the FEM-PrEP Study Group. 2012. Preexposure prophylaxis for HIV infection among African women. N Engl J Med 367:411–422. 10.1056/NEJMoa1202614. 22784040 PMC3687217

[9] Finlayson T, Cha S, Xia M, Trujillo L, Denson D, Prejean J, Kanny D, Wejnert C, National HIV Behavioral Surveillance Study Group. 2019. Changes in HIV preexposure prophylaxis awareness and use among men who have sex with men - 20 urban areas, 2014 and 2017. MMWR Morb Mortal Wkly Rep 68:597–603. 10.15585/mmwr.mm6827a1. 31298662 PMC6741853

[10] UNAIDS. 2014. The gap report. UNAIDS, Geneva, Switzerland. https://www.unaids.org/en/resources/documents/2014/20140716_UNAIDS_gap_report.

[11] Landovitz RJ, Donnell D, Clement M, Hanscom B, Cottle L, Coelho L, Cabello R, Chariyalestak S, Dunne E, Frank I, Gallardo J, Gaur A, Gonzalez P, Ha V, Hinojosa J, Kallas E, Kelley C, Losso M, Valdez Madruga J, Middelkoop K, Phanuphak N, Santos B, Sued O, Valencia HJ, Overton ET, Swaminathan S, del Rio C, Gulick R, Richardson P, Sullivan P, Piwowar-Manning E, Marzinke M, Marrazzo J, Daar E, Asmelash A, Anderson P, Eshleman S, Blanchette C, Lucas J, Psaros C, Safren S, Sugarman J, Scott H, Eron J, Fields S, Gomez-Feliciano K, Jennings A, Shin K, Rooney J, Spreen W, Margolis D, Rinehart A, Adeyeye A, Cohen M, McCauley M, Grinsztejn B, for the HPTN 083 Study Team. 2020. HPTN 083 final results: pre-exposure prophylaxis containing long-acting injectable cabotegravir is safe and highly effective for cisgender men and transgender women who have sex with men. Abstr 23rd International AIDS Conference, abstr OAXLB0101.

[12] Delany-Moretlwe S, Hughes J, Bock P, Gurrion S, Hunidzarira P, Kalonji D, Kayange N, Makhema J, Mandima P, Mathew C. 2021. Long acting injectable cabotegravir is safe and effective in preventing HIV infection in cisgender women: interim results from HPTN 084. Abstr HIV Research for Prevention, abstr HY01.02LB.

[13] Ding Y, Yan H, Ning Z, Cai X, Yang Y, Pan R, Zhou Y, Zheng H, Gao M, Rou K, Wu Z, He N. 2016. Low willingness and actual uptake of pre-exposure prophylaxis for HIV-1 prevention among men who have sex with men in Shanghai, China. Biosci Trends 10:113–119. 10.5582/bst.2016.01035. 27052151

[14] Liu J, Deng R, Lin B, Pan H, Gao Y, Dai J, Liang H, Huang A, Zhong X. 2021. Risk management on pre-exposure prophylaxis adherence of men who have sex with multiple men: a multicenter prospective cohort study. Risk Manag Healthc Policy 14:1749–1761. 10.2147/RMHP.S295114. 33953624 PMC8092636

[15] Peng L, Cao W, Gu J, Hao C, Li J, Wei D, Li J. 2019. Willingness to use and adhere to HIV pre-exposure prophylaxis (PrEP) among men who have sex with men (MSM) in China. Int J Environ Res Public Health 16:2620. 10.3390/ijerph16142620. PMC6678719 31340482

[16] Qu D, Zhong X, Xiao G, Dai J, Liang H, Huang A. 2018. Adherence to pre-exposure prophylaxis among men who have sex with men: a prospective cohort study. Int J Infect Dis 75:52–59. 10.1016/j.ijid.2018.08.006. 30125688

[17] Zhang Y, Cai C, Wang X, Li Y, Tang H, Ma J. 2020. Disproportionate increase of new diagnosis of HIV/AIDS infection by sex and age - China, 2007–2018. China CDC Wkly 2:69–74. 10.46234/ccdcw2020.020. 34594810 PMC8393105

[18] Zheng S. 2018. The growing threat of China's HIV epidemic. Lancet Public Health 3:e311. 10.1016/S2468-2667(18)30098-7. 29976325

[19] Akaba K, Kimura T, Sasaki A, Tanabe S, Ikegami T, Hashimoto M, Umeda H, Yoshida H, Umetsu K, Chiba H, Yuasa I, Hayasaka K. 1998. Neonatal hyperbilirubinemia and mutation of the bilirubin uridine diphosphate-glucuronosyltransferase gene: a common missense mutation among Japanese, Koreans and Chinese. Biochem Mol Biol Int 46:21–26. 10.1080/15216549800203512. 9784835

[20] Bowers GD, Culp A, Reese MJ, Tabolt G, Moss L, Piscitelli S, Huynh P, Wagner D, Ford SL, Gould EP, Pan R, Lou Y, Margolis DA, Spreen WR. 2016. Disposition and metabolism of cabotegravir: a comparison of biotransformation and excretion between different species and routes of administration in humans. Xenobiotica 46:147–162. 10.3109/00498254.2015.1060372. 26134155

[21] Patel P, Xue Z, King KS, Parham L, Ford S, Lou Y, Bakshi KK, Sutton K, Margolis D, Hughes AR, Spreen WR. 2020. Evaluation of the effect of UGT1A1 polymorphisms on the pharmacokinetics of oral and long-acting injectable cabotegravir. J Antimicrob Chemother 75:2240–2248. 10.1093/jac/dkaa147. 32361755 PMC7366207

[22] Barbarino JM, Haidar CE, Klein TE, Altman RB. 2014. PharmGKB summary: very important pharmacogene information for UGT1A1. Pharmacogenet Genomics 24:177–183. 10.1097/FPC.0000000000000024. 24492252 PMC4091838

[23] Han K, Baker M, Lovern M, Paul P, Xiong Y, Margolis DA, Spreen WR, Moore K, Ford SL. 2019. Population pharmacokinetics of cabotegravir in adult healthy subjects and HIV-1 infected patients following administration of oral tablet and long acting intramuscular injection. Abstr American Conference on Pharmacometrics, abstr W-052. 10.1111/bcp.15439 PMC9543358 35695476

[24] World Obesity Federation. 2021. Report card. China. World Obesity Federation, London, UK. https://data.worldobesity.org/country/china-42/report-card.pdf.

[25] World Obesity Federation. 2021. Report card. United States. World Obesity Federation, London, UK. https://data.worldobesity.org/country/united-states-227/report-card.pdf.

[26] Ford SL, Stancil BS, Markowitz M, Frank I, Grant RM, Mayer KH, Elion R, Goldstein D, Fisher C, Sobieszczyk ME, Gallant JE, Van Tieu H, Weinberg W, Margolis DA, Hudson KJ, Patel P, Rinehart AR, Smith KY, Spreen WR. 2016. ECLAIR study of cabotegravir (CAB) LA injections: characterization of safety and PK during the “PK tail” phase. Abstr HIV Research for Prevention, abstr OA12.06LB.

[27] Markowitz M, Frank I, Grant RM, Mayer KH, Elion R, Goldstein D, Fisher C, Sobieszczyk ME, Gallant JE, Van Tieu H, Weinberg W, Margolis DA, Hudson KJ, Stancil BS, Ford SL, Patel P, Gould E, Rinehart AR, Smith KY, Spreen WR. 2017. Safety and tolerability of long-acting cabotegravir injections in HIV-uninfected men (ECLAIR): a multicentre, double-blind, randomised, placebo-controlled, phase 2a trial. Lancet HIV 4:e331–e340. 10.1016/S2352-3018(17)30068-1. 28546090

[28] Landovitz RJ, Li S, Eron JJ, Jr, Grinsztejn B, Dawood H, Liu AY, Magnus M, Hosseinipour MC, Panchia R, Cottle L, Chau G, Richardson P, Marzinke MA, Eshleman SH, Kofron R, Adeyeye A, Burns D, Rinehart AR, Margolis D, Cohen MS, McCauley M, Hendrix CW. 2020. Tail-phase safety, tolerability, and pharmacokinetics of long-acting injectable cabotegravir in HIV-uninfected adults: a secondary analysis of the HPTN 077 trial. Lancet HIV 7:e472–e481. 10.1016/S2352-3018(20)30106-5. 32497491 PMC7859863

[29] Landovitz RJ, Li S, Grinsztejn B, Dawood H, Liu AY, Magnus M, Hosseinipour MC, Panchia R, Cottle L, Chau G, Richardson P, Marzinke MA, Hendrix CW, Eshleman SH, Zhang Y, Tolley E, Sugarman J, Kofron R, Adeyeye A, Burns D, Rinehart AR, Margolis D, Spreen WR, Cohen MS, McCauley M, Eron JJ. 2018. Safety, tolerability, and pharmacokinetics of long-acting injectable cabotegravir in low-risk HIV-uninfected individuals: HPTN 077, a phase 2a randomized controlled trial. PLoS Med 15:e1002690. 10.1371/journal.pmed.1002690. 30408115 PMC6224042

[30] Orkin C, Oka S, Philibert P, Brinson C, Bassa A, Gusev D, Degen O, González García J, Bernal Morell E, Tan DHS, D'Amico R, Dorey D, Griffith S, Thiagarajah S, St Clair M, Van Solingen-Ristea R, Crauwels H, Ford SL, Patel P, Chounta V, Vanveggel S, Cutrell A, Van Eygen V, Vandermeulen K, Margolis DA, Smith KY, Spreen WR. 2021. Long-acting cabotegravir plus rilpivirine for treatment in adults with HIV-1 infection: 96-week results of the randomised, open-label, phase 3 FLAIR study. Lancet HIV 8:e185–e196. 10.1016/S2352-3018(20)30340-4. 33794181

[31] Swindells S, Andrade-Villanueva J-F, Richmond GJ, Rizzardini G, Baumgarten A, Masiá M, Latiff G, Pokrovsky V, Bredeek F, Smith G, Cahn P, Kim Y-S, Ford SL, Talarico CL, Patel P, Chounta V, Crauwels H, Parys W, Vanveggel S, Mrus J, Huang J, Harrington CM, Hudson KJ, Margolis DA, Smith KY, Williams PE, Spreen WR. 2020. Long-acting cabotegravir and rilpivirine for maintenance of HIV-1 suppression. N Engl J Med 382:1112–1123. 10.1056/NEJMoa1904398. 32130809

[32] Spreen W, Ford SL, Chen S, Wilfret D, Margolis D, Gould E, Piscitelli S. 2014. GSK1265744 pharmacokinetics in plasma and tissue after single-dose long-acting injectable administration in healthy subjects. J Acquir Immune Defic Syndr 67:481–486. 10.1097/QAI.0000000000000301. 25140909

[33] Spreen W, Williams P, Margolis D, Ford SL, Crauwels H, Lou Y, Gould E, Stevens M, Piscitelli S. 2014. Pharmacokinetics, safety, and tolerability with repeat doses of GSK1265744 and rilpivirine (TMC278) long-acting nanosuspensions in healthy adults. J Acquir Immune Defic Syndr 67:487–492. 10.1097/QAI.0000000000000365. 25473882

[34] Murray MI, Markowitz M, Frank I, Grant RM, Mayer KH, Hudson KJ, Stancil BS, Ford SL, Patel P, Rinehart AR, Spreen WR, Margolis DA. 2018. Satisfaction and acceptability of cabotegravir long-acting injectable suspension for prevention of HIV: patient perspectives from the ECLAIR trial. HIV Clin Trials 19:129–138. 10.1080/15284336.2018.1511346. 30445896

[35] Bradley C. 2020. The study medication satisfaction questionnaire (SMSQ) user guidelines. Health Psychology Research Ltd., Egham, UK. https://www.healthpsychologyresearch.com/sites/default/files/guidelines/SMSQ%20Summary%202Apr20.pdf.

[36] Andrews CD, Spreen WR, Mohri H, Moss L, Ford S, Gettie A, Russell-Lodrigue K, Bohm RP, Cheng-Mayer C, Hong Z, Markowitz M, Ho DD. 2014. Long-acting integrase inhibitor protects macaques from intrarectal simian/human immunodeficiency virus. Science 343:1151–1154. 10.1126/science.1248707. 24594934 PMC4308974

[37] Andrews CD, Yueh YL, Spreen WR, St Bernard L, Boente-Carrera M, Rodriguez K, Gettie A, Russell-Lodrigue K, Blanchard J, Ford S, Mohri H, Cheng-Mayer C, Hong Z, Ho DD, Markowitz M. 2015. A long-acting integrase inhibitor protects female macaques from repeated high-dose intravaginal SHIV challenge. Sci Transl Med 7:270ra4. 10.1126/scitranslmed.3010298. PMC4449736 25589630

